# Advancements in Transparent Conductive Oxides for Photoelectrochemical Applications

**DOI:** 10.3390/nano14070591

**Published:** 2024-03-27

**Authors:** He Wen, Bo Weng, Bing Wang, Wenbo Xiao, Xiao Liu, Yiming Wang, Menglong Zhang, Haowei Huang

**Affiliations:** 1 School of Semiconductor Science and Technology, South China Normal University, Foshan 528225, China; hewen@m.scnu.edu.cn (H.W.); bingwang@m.scnu.edu.cn (B.W.); liuxiao@m.scnu.edu.cn (X.L.); yimingwang@m.scnu.edu.cn (Y.W.); 2 Institute of Urban Environment, Chinese Academy of Sciences, Xiamen 361021, China; bweng@iue.ac.cn; 3 Key Laboratory of Nondestructive Test, Ministry of Education, Nanchang Hangkong University, Nanchang 330063, China; xiaowenbo1570@163.com; 4 Zhejiang Xinke Semiconductor Co., Ltd., Hangzhou 311421, China; 5 Centre for Membrane Separations, Adsorption, Catalysis and Spectroscopy for Sustainable Solutions (cMACS), Department of Microbial and Molecular Systems, KU Leuven, 3001 Leuven, Belgium; haowei.huang@kuleuven.be

**Keywords:** TCO materials, PEC reaction, nanostructure, solar energy, photoelectrode

## Abstract

**Highlights:**

**What are the main findings?**

- Doping can improve TCO’s electrical conductivity whilst minimizing any significant loss in their optical transmission.
- Further modification techniques increase the surface energy of TCO, reduce particles and defects, and improve electrical conductivity.

**What is the implication of the main finding?**

- TCO materials show promise for enhancing the efficiency and effectiveness of photoelectrochemical devices.
- Advancements for TCO materials lead to the development of photoelectrochemical conversion technology.

**Abstract:**

Photoelectrochemical cells (PECs) are an important technology for converting solar energy, which has experienced rapid development in recent decades. Transparent conductive oxides (TCOs) are also gaining increasing attention due to their crucial role in PEC reactions. This review comprehensively delves into the significance of TCO materials in PEC devices. Starting from an in-depth analysis of various TCO materials, this review discusses the properties, fabrication techniques, and challenges associated with these TCO materials. Next, we highlight several cost-effective, simple, and environmentally friendly methods, such as element doping, plasma treatment, hot isostatic pressing, and carbon nanotube modification, to enhance the transparency and conductivity of TCO materials. Despite significant progress in the development of TCO materials for PEC applications, we at last point out that the future research should focus on enhancing transparency and conductivity, formulating advanced theories to understand structure–property relationships, and integrating multiple modification strategies to further improve the performance of TCO materials in PEC devices.

## 1. Introduction

With the fast development of human society in the last hundred years, energy demand is soaring up. Currently, fossil fuels are the main energy source in our society. However, the rising costs, depletion of nonrenewable resources, and environmental pollution associated with fossil fuels underscore the urgent need for sustainable and clean energy solutions. The development of sustainable and clean energy has become a crucial task in recent years [1]. Solar energy is abundant, renewable, and clean, making it a promising solution to meet the global energy challenge, which has garnered widespread attention from various countries and regions [2].

Photoelectrochemical cells (PECs) are an efficient approach to convert, utilize, and store solar energy, which plays a crucial role in helping to transition us away from the burning of fossil fuels and to reduce carbon emissions [3]. Photoelectrochemical cells (PECs), consist of one or two semiconductor photoelectrodes, as well as a counter electrode immersed in an electrolyte. In photoelectrodes, in addition to semiconductors acting as light absorbers to collect solar energy and generate charge carriers, transparent conductive oxides (TCOs) are another key component. Due to their transparency and conductivity, TCO materials are often used as a charge transport layer to support and immobilize semiconductor photoelectrocatalysts. Besides PEC devices, TCOs are wildly used in optoelectronic devices such as solar cells, LEDs, and optical sensors. Zinc oxide (ZnO) [4], Al-doped zinc oxide (AZO) [5], indium oxide [6], tin-doped indium oxide (ITO) [7], and fluorine-doped tin oxide (FTO) [8] are typical substrate materials for supporting PEC reactions.

**Figure 1:**
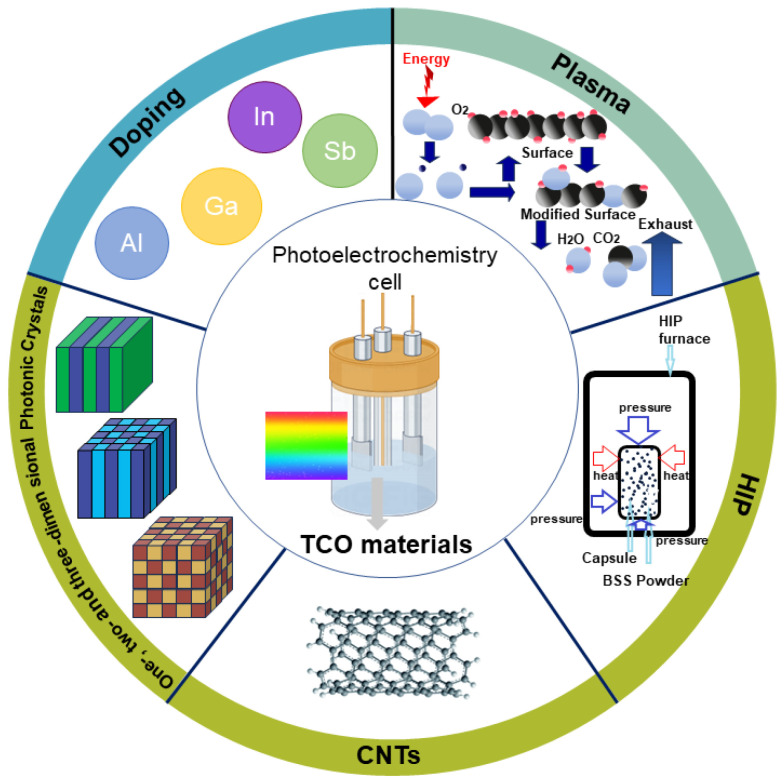
Summary of transparent conductive oxides development for photoelectrochemical application.

The recognition of TCO materials’ pivotal role in PECs underscores the necessity for a comprehensive review to comprehend their cutting-edge status in the realm of PECs. This review is dedicated to an exhaustive exploration of TCO materials, encompassing intrinsic properties, fabrication techniques, extant challenges, and state-of-the-art optimization methodologies, as shown in Figure 1. This paper commences by elucidating the fundamental properties that render TCO materials indispensable in facilitating PEC reactions. Subsequently, we delve into recent advancements propelling TCO materials to the forefront in supporting and enhancing PEC reactions. Next, techniques employed in TCO material fabrication will be meticulously examined, establishing a foundation for understanding synthesis intricacies. In the fourth section, we explore modification techniques transcending conventional boundaries, augmenting TCO properties through cutting-edge methods like doping element technology, plasma radio frequency technology, hot isostatic pressing, TCO substrate carbon nanotube technology, and photonic crystals. 

This review provides a comprehensive overview and analysis of the use of TCO materials in PEC reactions, from basic properties, recent advances, fabrication techniques, and modification technologies to future challenges. The aim is to provide the reader with a comprehensive understanding of the subject. Additionally, this article not only examines the fundamental characteristics of TCO materials but also explores the latest research advancements and state-of-the-art modification techniques. For instance, the review discusses advanced techniques such as doping element technology, plasma radio frequency technology, thermal isostatic pressure technology, TCO substrate carbon nanotube technology, and photonic crystals, all of which are current areas of research interest. The concluding section presents the current persistent challenges as a basis for future research.

## 2. TCO Materials

As a substrate for photoelectrodes, TCO materials have the characteristics of high electrical conductivity, high optical clarity, stability, high compatibility, low resistivity, high optical transmittance in the visible range, and high optical reflectivity in the infrared range [9]. Their transparency allows light to pass through the material to the photocatalysts, allowing them to transmit light efficiently in photoelectrochemical devices, ensuring maximum utilization of light energy. Aside from transparency, TCO materials must have selective conductivity for electrons or holes. Indium tin oxide, fluorine-doped SnO_2_, aluminum-doped ZnO, etc., can act as electrode materials, providing a reliable electron transport channel in photoelectrochemical devices. Light shines on the electrode–electrolyte solution system, resulting in charge separation and redox reactions, leading to the conversion of solar energy into electrical or chemical energy. In photoelectrochemical reactions, the effective separation of photogenerated electrons and holes is the key to achieving efficient energy conversion. If electrons and holes are not effectively separated, they will rapidly complex, resulting in energy loss. Electron- or hole-selective conductivity can facilitate the effective separation of carriers, thereby improving the energy conversion efficiency of photoelectrochemical devices. TCOs are doped metal oxides commonly used in optoelectronic devices, such as flat panel displays and photovoltaics, including inorganic, organic, and dye-sensitized solar cells. Generally, TCO plays two roles in solar cells: (1) the TCO film collects charge carriers and acts as an electrode and (2) the front TCO film also acts as an antireflective coating [10]. For example, dye-sensitized solar cells are mainly composed of several parts, such as a nanoporous semiconductor film, dye sensitizer, a redox electrolyte, a counter electrode, and a conductive substrate. Among them, the nanoporous semiconductor film is usually composed of TiO_2_, SnO_2_, ZnO, etc., which are aggregated on a glass plate with a transparent conductive film as the cathode of DSC. The counter electrode acts as a reduction catalyst and is usually platinum coated on glass with a transparent conductive film. The sensitized dye is adsorbed on the nanoporous titanium dioxide membrane surface [11]. These films are usually fabricated with polycrystalline or amorphous microstructures. Electrode materials with over 80% transmittance of incident light and electrical conductivities higher than 10^3^ S/cm are typically used for efficient carrier transport. For thin-film electrodes in solar cells, it is generally recommended that TCOs have a minimum carrier concentration of around 1020 cm^−3^ to achieve low resistivity and a bandgap greater than 3.2 eV to avoid light absorption across most of the solar spectra [12]. The mobility of these films is typically limited by ionized impurity scattering due to the large amount of ionized dopant atoms, resulting in a mobility of around 40 cm^2^/(V·s) for the best performing TCOs [13]. Table 1 presents performance values such as electrical conductivity, light transmittance, thermal and chemical stability, versatility, and E_g_ for various TCO materials.

The evaluation criteria for TCOs in photoelectrochemical applications should focus on the relevant performance indicators listed in the table. Firstly, optical properties are crucial. TCOs must have high transparency in the visible spectral range to allow light to pass through and be absorbed by the photoactive material. This is typically assessed by measuring transmittance and reflectance, with the aim of maximizing the former and minimizing the latter. The reflectivity of TCO is usually low in the visible range, but the exact value depends on the thickness of the film and the preparation process. Next, conductivity should be high to ensure efficient charge collection and transport. Following this, the bandgap energy of the TCO should be optimized to allow for efficient charge separation and prevent unwanted light absorption, which could reduce the efficiency of the underlying photoactive material. Chemical stability is crucial for TCOs to be used in photoelectrochemical cells. They must be able to withstand the operating conditions, including resistance to corrosion in water-based electrolytes and photo corrosion. Additionally, it is important to consider the materials’ mechanical stability, cost, compatibility, and environmental impact.

**Figure 2:**
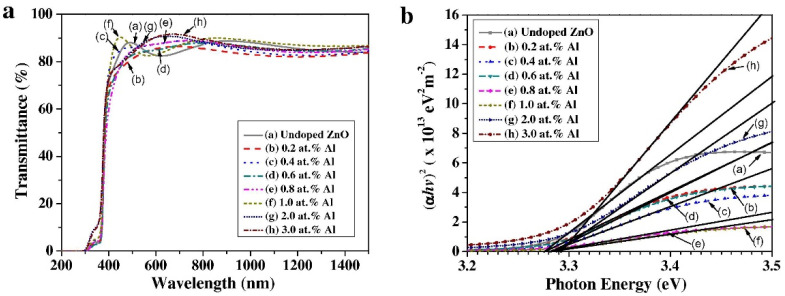
(**a**) Transmission spectra of undoped and Al-doped ZnO thin films and (**b**) estimation of optical band gap energy of undoped and Al-doped ZnO thin films. Reproduced with permission of Ref. [34], Copyright of © 2010 Optical Materials.

Zinc oxide (ZnO), arranged in a hexagonal close packing (HCP) structure, is a common TCO material; its electron μ_n_ is 130–440 cm^2^V^−1^s^−1^, and its hole μ_p_ is 0.1–50 cm^2^V^−1^s^−1^ [30]. Owing to its abundant and economical availability, high electron mobility, broad band gap, and excellent transparency, ZnO holds immense potential for various emerging applications, including transparent electrodes, liquid crystal displays, light-emitting diodes, energy-conserving or thermally protective windows, and thin-film transistors. ZnO nanocrystalline films are widely used as electron transport layers (ETL) in quantum dot light-emitting diodes (QLEDs) due to their excellent electron transport properties. The role of ETL is to help electrons to be injected from the cathode into the light-emitting layer and to be transported efficiently through the light-emitting layer, thus improving the luminous efficiency and performance of LEDs [31]. As the substrate of the photoelectrode, due to the surface stability of the polar surface of ZnO, the internal electrostatic field near the surface of it will gradually decrease to zero. Therefore, the interfacial charge separation is mainly determined by the interfacial electric field in the space charge region formed by the Fermi level equilibrium between the ZnO and the electrolyte solution. However, the difference in the bulk charge transfer efficiency of ZnO is much larger than that at the interface, suggesting that bulk charge transfer may play a more important role in determining the total charge transfer. Nonetheless, pure ZnO exhibits high resistance, and its electrical stability diminishes when ambient temperatures exceed 150 °C. ZnO can have a reduction potential window starting at around 0 V vs. a reversible hydrogen electrode (RHE), but it is susceptible to reduction at relatively positive potentials compared to some other TCOs, which limits its use in certain electrochemical applications. To obtain low resistance, trace elements can be doped into ZnO. It is relatively easy to achieve n-type doping of ZnO due to the fact that the Fermi energy levels of ZnO are pinned much higher than the bottom of its conduction band. The dopant elements include the main group III elements aluminum (Al), boron (B), gallium (Ga), and indium (In); the subgroup III elements scandium (Sc) and yttrium (Y); and the main group IV elements silicon (Si), germanium (Ge), and tin (Sn). These elements are doped to take the place of Zn, releasing unpaired s valence electrons. Aluminum-doped ZnO (AZO) also has an HCP geometric structure [32], but Al partially replaces the Zn in the six-membered ring. Al doping introduces extra free electrons, leading to a higher carrier density and better conductivity which is beneficial for the migration of carriers generated in the photoelectrocatalyst, resulting in an elevated photocurrent [33]. AZO also has a reduction potential window starting at around 0 V vs. an RHE, similar to ZnO. Figure 2 shows the transmission spectra and estimation of the optical band gap energy of undoped and Al-doped ZnO thin films. Figure 2a shows that all films exhibit high transmittance >80% in the wavelength range of 400–1500 nm and have a sharp absorption edge near the wavelength of 370 nm, while from Figure 2b, it can be concluded that films with higher Al concentrations have a wider optical bandgap energy compared to undoped ZnO [34]. K. Govatsi et al. [35] fabricated grown Al-doped ZnO NW arrays with doping concentrations ranging from 0 to 2.0 at. % Al. The prepared samples were used as photoanodes for water decomposition, where the samples were excited by sunlight to produce photogenerated electrons and holes, which were partially separated by applied bias drive for water oxidation at the anode of the electrolytic cell and for proton reduction reaction at the cathode. They observed a 2-fold increase in specific area current density (flip-flop) and a 3–4-fold increase in charge carrier density when zinc oxide was doped with aluminum ions. However, the electrical conductivity of AZO materials is easily affected by environmental factors, such as temperature and humidity, which can lead to irreversible changes and ultimately impact their efficacy [36]. Another key property of AZO is its high transparency, especially in the visible range. This transparency is due to its large band gap, which allows visible light to pass easily through the material without being absorbed. Furthermore, the band gap width of AZO can be precisely controlled by adjusting the Al doping concentration. Increasing the amount of doping results in a widening of the bandgap. This property makes AZO an ideal material for devices such as displays and solar cells, which require high light transmission to improve efficiency and display quality. Y. Bouznit et al. [37] found a significant increase in photocurrent at a 2% Al doping level as compared to pure ZnO, indicating a higher efficiency of photogenerated electron–hole pair separation with Al doping. When the semiconductor absorbs energy in the form of light greater than its Eg, the conductivity of the material increases with the generation of electron–hole pairs. On the other hand, different phenomena on the electrode surface such as desorption and oxygen adsorption can also control the photocurrent generation process. Meanwhile, the addition of aluminum can improve the thermal stability of ZnO beyond 500 °C [38]. In addition, AZO exhibits better chemical stability and durability, rendering it viable for sustained usage in photoelectrochemical reaction systems.

Indium oxide (In_2_O_3_) and tin-doped indium oxide (ITO) have an HCP crystal structure [39], with the possibility of slight alterations in the crystal structure due to the introduction of Sn^2+^ doping. In_2_O_3_ has a reduction potential window similar to ITO and IZO, starting at around 0 V vs. an RHE and extending to slightly negative potentials. Compared with In_2_O_3_, ITO is widely used in photoelectrodes and other photoelectric devices, because Sn doping enhances the conductivity and optical transparency of In_2_O_3_ [40]. Sn^2+^ has a similar ionic radius as In^3+^, which avoids obvious lattice distortion during the substitution process. At the same time, oxygen vacancies are generated, producing free electrons that contribute to the conductivity of the material. Therefore, ITO exhibits a low resistivity on the order of 10^−4^ Ω · cm. On the other hand, ITO is a wide bandgap film material with a bandgap range of 3.5–4.3 eV. In the visible light region, the transmittance of ITO films is excellent. However, in the UV region, below 330 nm, most photonics can be absorbed by the ITO [41]. At the same time, in the near-infrared region, the light transmittance of the ITO is also very low due to the plasma oscillation phenomenon of the charge carriers. ITO is known for its high stability, but the reduction potential window is generally less negative than that of FTO due to the more noble nature of indium. The window typically starts at around 0 V vs. RHE and extends to slightly negative potentials. However, high doping concentration of Sn^2+^ would lead to lower light transmittance. Usually, the optimal doping concentration of Sn is about 5–10% [41]. ITO, as an electrode, has superiority over metal electrodes commonly used for electroanalysis due to its low cost, low electrochemical background response, wide working potential window, easy surface functionalization, and commercial availability [42,43]. Unfortunately, the production of ITO is hindered by the severe shortage of indium, high production cost, and the brittleness of the material itself [44]. Thus, fluorine-doped tin oxide (FTO) has emerged as the most promising substitute for ITO with superior thermal and chemical stability. Compared to ITO electrodes, FTO electrodes are more climate-stable and temperature-resistant, and are chemically inert, mechanically resistant, and highly resistant to physical abrasion, and the reduction potential window typically extends from around 0 V vs. an RHE to negative potentials. They are used to make transparent conductive coatings for touchscreens, flat-panel screens, airplane cockpit windows, and plasma displays, and thin oxide layers are used in the production of organic light-emitting diodes (OLEDs) and solar cells [45]. Jesse D. Benck et al. [46] found experimentally that the ITO electrode (2 V) has the narrowest potential range in 0.5 M Na_2_SO_4_. During anodic polarization, the substrate undergoes slow degradation due to oxidation of the ITO oxide layer. On the other hand, the potential range of the FTO electrode is 0.6 V wider than that of the ITO electrode, which proves that this material has a better chemical stability in 0.5 M Na_2_SO_4_. The reactions on the FTO electrode related to the electrolysis of the supporting electrolyte are similar to those of the ITO electrode, but with a much lower signal intensity. When preparing the FTO film through radio frequency magnetron sputtering, defects arise during the transition process of the FTO film from an amorphous to a nanocrystalline structure [47]. The carrier mobility of the FTO film, which was annealed at 600 °C, is twice that of the unannealed sample. Additionally, the correlation between positron annihilation and the Hall effect demonstrates the significance of defect scattering in determining the charge carrier mobility [48].

## 3. Fabrication of TCO Films

**Figure 3:**
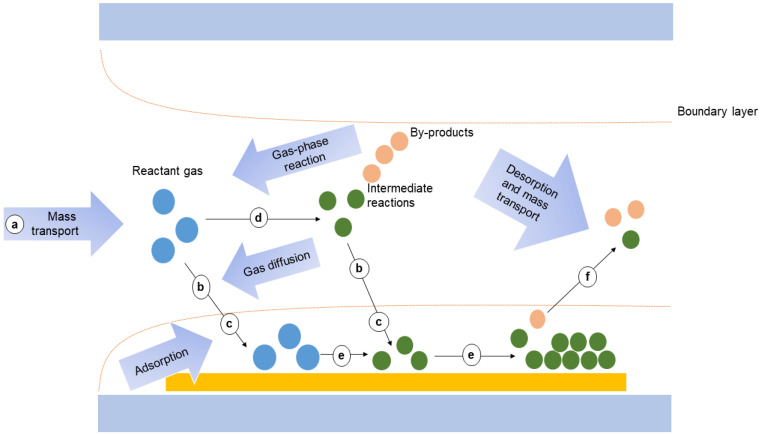
Schematic of the general elementary steps of a typical CVD process. First, reactant gases (blue circles) are transported into the reactor (step a). Then, there are two possible routes for the reactant gases: directly diffusing through the boundary layer (step b) and adsorbing onto the substrate (step c) or forming intermediate reactants (green circles) and byproducts (red circles) via the gas-phase reaction (step d) and being deposited onto the substrate via diffusion (step b) and adsorption (step c). Surface diffusion and heterogeneous reactions (step e) take place on the surface of substrate before the formation of thin films or coatings. Finally, byproducts and unreacted species are desorbed from the surface and forced out of the reactor as exhausts (step f). CVD, chemical vapor deposition. Reproduced with permission of Ref. [58], Copyright of © 2021 Nature Reviews Methods Primers.

TCO films can be deposited onto a substrate using a variety of deposition techniques, including chemical vapor deposition (CVD) [49], metalorganic chemical vapor deposition (MOCVD) [50], metal–organic molecular-beam deposition (MOMBD) [51], solution deposition [52], spray pyrolysis [53], sol–gel method [54], pulsed laser deposition (PLD) [55], magnetron sputtering (MS) [56], and so on. Moreover, among these methods, CVD is a vacuum deposition method which is widely used for synthesizing high-quality solid materials with superior performance [57]. The process is often used in the semiconductor industry to produce thin films, and the schematic of the general elementary steps of a typical CVD process is shown in Figure 3 [58].

This approach is markedly advantageous for atomic layer deposition. Firstly, CVD provides precise control over film composition and doping levels. Secondly, it allows high-quality, well-structured films to grow. Thirdly, CVD maintains excellent uniformity and repeatability, particularly over large areas. Fourthly, the technique facilitates film growth at relatively low temperatures and pressures, promoting substrate preservation and potentially reducing energy costs. Fifthly, CVD techniques often exhibit higher growth rates, enabling the rapid fabrication of large-area films and enhancing overall production efficiency [59]. These attributes collectively make CVD a preferred choice for depositing TCO films with enhanced control, quality, and efficiency. Thin films deposited through CVD have wide-ranging applications across different industries. Depending on the operating conditions, CVD can take a variety of forms, such as atmospheric pressure CVD (APCVD), low-pressure CVD (LPCVD), and ultra-high vacuum CVD (UHVCVD). This technique has revolutionized materials science, promoting the development of advanced electronic, optical, and mechanical devices with unmatched precision, uniformity, and quality.

Low-pressure chemical vapor deposition (LPCVD) operates at pressures below atmospheric pressure, typically ranging from 10 mTorr to 100 mTorr [60]. In the LPCVD process, gas and metal–organic precursors are introduced into a reaction chamber, where they undergo decomposition and subsequently deposit onto the substrate surface under elevated temperatures. This technique offers precise control over the composition, crystal structure, and thickness of the deposited materials. LPCVD is frequently employed in the creation of transparent conductive oxides (TCOs) and the fabrication of semiconductor, optical, and electronic devices. The method excels in producing highly transparent and conductive films characterized by low resistivity, high carrier concentration, and excellent microscopic uniformity. These attributes collectively enhance the electrical conductivity and transparency of the films, making LPCVD a valuable technique in the production of advanced materials for various applications. To cite an instance, Uematsu et al. [61] explored that LPCVD is used for depositing boron-doped ZnO, which holds promise as a TCO for thin-film silicon solar cells. Another example of LPCVD is provided by J. Meier et al. [62] who have shown that optical characterizations have demonstrated that ZnO after LPCVD has a stronger light-trapping effect than SnO_2_. This advantage, in conjunction with the low cost of LPCVD ZnO, has facilitated its utilization as a substitution for TCO material in thin-film silicon solar cells. Specifically, stabilized a-Si:H p-i-n solar cell efficiencies of up to 9% have been achieved using LPCVD ZnO, which demonstrates its potential as a cost-effective and efficient alternative to traditional thin-film silicon solar cell materials.

In comparison to low-pressure chemical vapor deposition (LPCVD), atmospheric pressure chemical vapor deposition (APCVD) stands out due to its simplicity in operation, equipment, and lower cost [63]. Specifically, in the preparation of transparent conductive oxide (TCO) materials, APCVD offers the capability to adjust the microstructure as well as the physical and chemical properties of the film by controlling key reaction parameters, such as reaction temperature and the amount of oxide precursor. This precise control allows for the optimization of performance. An illustrative example of APCVD’s effectiveness is found in the work of Dagkaldiran, Ü. et al. [64], where a novel process for depositing fluorine-doped tin oxide (FTO) thin films was developed using APCVD. This technique was tailored to meet industrial requirements such as rapid processing times, scalability to larger substrate widths, and reduced overall cost. The study puts forth a scalable and cost-effective method for FTO thin film production that holds promise for meeting the demands of large-scale industrial production of TCO materials [65].

Ultra-high vacuum chemical vapor deposition (UHVCVD) is a vital method for the preparation of high-quality thin-film materials, with operational pressures typically less than 10^−10^ Pa [58]. UHVCVD employs a high vacuum environment where gases containing metal elements, oxygen, and nitrogen are introduced into a reaction chamber and then heated to a sufficiently high temperature to decompose and deposit the required thin-film material onto the substrate surface. Compared to conventional CVD, UHVCVD allows for the management of the thin-film microstructure, thickness, and defect density, resulting in superior-quality materials [66].

Although CVD has many advantages, it also has some obvious disadvantages. CVD must be carried out under high-temperature conditions, which can cause thermal damage or introduce impurities that affect material properties. Additionally, the high cost of CVD makes it unsuitable for mass production, and substrate pretreatment adds complexity to the preparation.

In addition, magnetron sputtering, one of the most commonly used techniques to synthetize TCO films, is a physical vapor deposition technology involving plasma, which can achieve high efficiency, low temperature, and low damage by introducing a magnetic field on the surface of the target cathode and using the magnetic field to restrict the charged particles to increase the plasma density and increase the sputtering rate. The principle is to use high-energy particles to bombard the solid target, so that the atoms or molecules on the surface of the target are sputtered out and deposited on the substrate surface to form a thin film. The properties of DC-sputtered thin films are influenced by applied DC power, sputtering pressure and atmosphere, target-to-substrate distance, substrate temperature, and film thickness [67]. Furthermore, in the case of ZnO:Al films, the use of ceramic targets sintered from composite ZnO–Al_2_O_3_ powders (so-called AZO targets) generally offers an easier control and a major reproducibility of both sputtering process and film properties with respect to reactive cosputtering from Zn and Al metallic targets.

Tsubasa Ide et al. [68] developed a cross-shaped magnetized RF sputtering plasma source for uniform circular target utilization. The cross-shaped plasma was achieved by inducing a linear E × B_r_ drift motion using a cruciform arrangement of neodymium magnets. The electrons are strongly magnetized by the Hall parameter at a magnitude 20 times that of the ions. Strong cross-shaped plasma discharges were observed. The ion saturation current was measured at various radial positions using a Langmuir probe, and it decreased from the center to the outer area of the target. The target utilization percentage increased from 73.6% to 86.3% when Fe pole pieces were incorporated on the neodymium magnets.

However, the magnetron sputtering method also has certain shortcomings, such as that the sputtering process temperature rise will produce some damage to the substrate, the rate of vacuum coating is low, and the required air pressure is high, in addition to the fact that the sputtering equipment system is complex, expensive, and so on. Therefore, additional research is required to enhance the transparency and conductivity of TCO materials using cost-effective, simple, efficient, and environmentally friendly fabrication methods.

## 4. Modification Techniques for Improving the Properties of TCO Materials

As previously noted, TCO materials exhibit highly favorable characteristics such as high transmittance, low resistivity, and excellent chemical stability, making them advantageous for PEC devices. However, a significant challenge arises from the inherent trade-off between conductivity and transparency, posing a notable impediment for TCO materials. Additionally, some TCO materials may display erratic behavior at elevated temperatures, causing disruptions in PEC reactions. Furthermore, specific TCO materials may exhibit high transmittance only for selective wavelengths of light, limiting their utility in certain PEC reactions. Moreover, certain TCO materials face restricted commercial application due to their high cost. The intricate growth methods for TCO materials can escalate production and engineering costs, posing challenges in regulating film thickness and lattice structure. These limitations have the potential to compromise the performance of TCO materials in PEC reactions [69]. To address these challenges, this section provides an overview of current modification technologies employed to enhance the performance of TCO materials. The exploration of these modification technologies serves as a valuable reference for subsequent industrial development, aiming to overcome the inherent limitations associated with TCO materials in the context of PEC reactions.

### 4.1. Element Doping

In the field of TCO materials, doping is a frequently used technique to improve their electrical conductivity whilst minimizing any significant loss in their optical transmission [70]. Doping involves introducing specific impurities, known as ‘dopants’ into the crystal lattice of the TCO material, which can increase the concentration of free electrons (for n-type doping) or holes (for p-type doping) in the semiconductor, thereby improving the material’s electrical conductivity. For example, n-type silicon semiconductors doped with a group V element such as phosphorus provide additional electrons to act as carriers, while p-type silicon semiconductors doped with a group III element such as boron increase the number of holes, thereby altering their electronic properties. Various techniques can be employed for doping, including cation, anion, and compound doping. Cation doping is frequently utilized to regulate TCO properties by incorporating elements such as Al, In, and Zn to enhance conductivity [71]. Additionally, improvements in conductivity can be achieved through anion doping. Introducing two or more elements simultaneously (compound doping) can also further enhance the conductivity of TCO.

For the design and manufacture of ZnO-based TCOs, doping is a pivotal concern. ZnO films are doped with some elements, such as In (from Group III elements) [72], Al (from Group III elements) [73], Ga (from Group III elements) [74], F (from Group VII elements) [75], Si (from Group IV elements) [76], etc. Among them, Al-doped ZnO and Ga-doped ZnO have been the most researched and analyzed. Al-doped ZnO has been introduced above. Ga is a Group IIIA element situated in the periodic table, and it has similar electronic properties as aluminum (Al). Ga has a smaller ionic radius than Zn but a similar ionic radius to Al. The Ga^3+^ ion radius is 0.062 nm, rendering it suitable for doping ZnO materials and enhancing their electrical properties. When Ga^3+^ ions are incorporated into the ZnO lattice, the smaller Ga^3+^ ions can substitute for Zn^2+^ ions. As a result, the substitution of Zn^2+^ ions with Ga^3+^ ions results in minimal lattice deformations in the ZnO material, even at higher doping concentrations. The minimal lattice deformations could reduce lattice defects and strain in the crystal structure, bringing about improved electrical properties in the Ga-doped ZnO thin films [77]. Zhifang Zhou et al. inserted homogeneous ZnO buffer layers of different thicknesses between the sapphire substrate and the GZO films and investigated their effect on TE performance. The thin ZnO interlayer (10 nm) effectively reduces the lattice mismatch of the GZO film and improves the carrier mobility, which greatly enhances the conductivity. At the same time, energy filtering occurs at the interface between GZO and ZnO, resulting in a relatively high effective density of states (DOS) and maintaining a high Seebeck coefficient compared to unbuffered GZO films. Therefore, the GZO film with a 10 nm thick ZnO buffer layer has a high power factor value of 449 μW m^−1^ K^−2^ at 623 K. The study provides a simple and effective way to optimize the TE properties of oxide films: synergistically improving their carrier mobility and increasing their effective mass [77].

**Figure 4:**
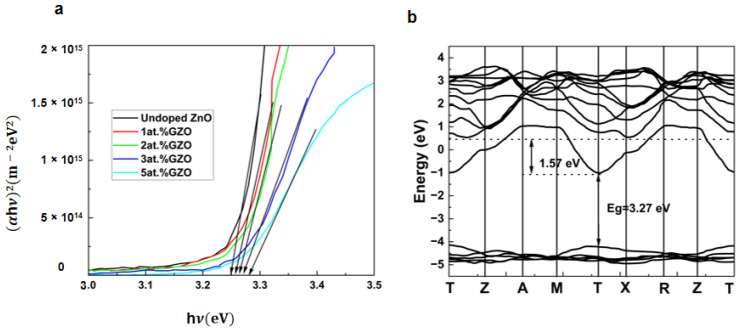
(**a**) Variation (αhν)^2^ of versus (hν) for the undoped ZnO and GZO thin films, reproduced with permission from Ref. [79], Copyright of © 2016 Ceramics International; (**b**) Band structure diagram, evaluated for bulk in SnO_2_ : 4 at% Sb, Reproduced with permission of Ref. [84], Copyright of © 2013 Applied Surface Science.

Furthermore, gallium doping can introduce acceptor levels in the ZnO bandgap, leading to a decrease in donor impurity concentration and an improvement in the p-type characteristics of ZnO. As a result, gallium doping enhances the electrical conductivity and carrier concentration of ZnO materials. Wang et al. [78] produced Ga-doped ZnO powder with an exceptional conductivity level of 300 $\Omega^{-1}$cm^−1^ at 25 °C, which is more than 1000 times higher than any previously reported values. These high values were measured at room temperature, whereas other studies of doped zinc oxide used measuring temperatures above 700 °C to obtain significant conductivity. They attribute their higher conductivity to their high synthesis temperatures and highly reducing conditions in closed systems. Their highly conducting samples have been equilibrated with metal vapors (Zn + Ga) at a pressure considerably in excess of 1 atm. Gallium doping in ZnO can also improve the optical properties of the material, such as increasing the bandgap energy. Muchuweni, E. et al. [79] investigated the impact of gallium doping on the structural, optical, and electrical characteristics of zinc oxide thin films created using spray pyrolysis. They discovered that introducing Ga resulted in an increment in the E_g_ value from 3.26 eV to 3.30 eV. Figure 4a illustrates the bandgap change in the undoped ZnO and GZO thin films. Gallium doping also leads to an increase in the full-width average and dislocation density as well as a decrease in the average grain size. All GZO films showed relatively high transparencies (~70–85%) in the visible region compared to the undoped ZnO films. According to the Burstein–Moss effect, the optical band gap shifted to shorter wavelengths with gallium doping, from 3.26 to 3.30 eV. An increase in Erbach energy was observed with the addition of gallium, indicating an increase in structural disorder and defects. In addition to enhancing electrical and optical properties, gallium doping in ZnO can also improve the chemical stability and increase its resistance to environmental degradation. This is because gallium oxide (Ga_2_O_3_) is more thermodynamically stable and less reactive than ZnO. The surface of Ga-doped ZnO thin films is protected from chemical degradation, and durability is increased due to the presence of Ga_2_O_3_.

Cation doping also receives significant attention with respect to SnO_2_-based TCO materials attributed to their availability and potential applications. Several dopants, including indium (In), antimony (Sb), titanium (Ti), niobium (Nb), and cerium (Ce), have been reported to enhance the performance of SnO_2_ materials [80,81,82]. Among these dopants, In and Sb are the most frequently used and have been demonstrated to enhance the transparency and conductivity of SnO_2_. Sb-doped SnO_2_ (ATO) has gained remarkable attention owing to its low cost, high transmittance in the visible light range, and high absorption in the NIR region [83]. The doping of Sb^5+^ ions into the SnO_2_ lattice creates an SbO_4_ tetrahedron, which increases the electron concentration and decreases the band gap of the material. Floriano, E. A. et al. [84] doped tin dioxide (SnO_2_) with pentavalent Sb^5+^ ions through a sol–gel–dip technique, resulting in enhanced conductivity of the material, as Sb^5+^ replaces Sn^4+^ in the matrix, which promotes an increase in the electron density in the CB. The energy band structure of bulk SnO_2_ : 4 at% Sb is presented in Figure 4b, where $E_{F}$ denotes the Fermi level energy. The diagram reveals that both the VB top and CB bottom are situated along the same direction. When the concentration of Sb ions is high, the energy levels become degenerate, consequently leading to the elevation of the Fermi level above the CB minimum. These findings align with the anticipated behavior of semiconductors with high doping levels. Mazloom et al. [85] successfully deposited ATO films on glass substrates using the sol–gel–dip-coating technique and obtained fiber-like stripe thin films that exhibited a high transmittance in the visible range of light and superior conductivity.

In addition to the doping of these common TCO materials, there are many examples of doping in TCO materials intended to enhance their performance, which will be introduced in detail in Table 2.

From the above studies, doped TCO materials have obvious advantages over undoped materials in terms of increased conductivity, enhanced optical properties, improved simplex characteristics, and extended application areas. Hence, if the conductive or photoelectrochemical properties of the TCO materials under study are defective, we can try to solve them by introducing doping. In addition, it is also clear that we need advanced theories to study the reaction mechanisms and gain a comprehensive understanding of the structure–property relationships and carrier transfer mechanisms of TCO materials.

### 4.2. Plasma for TCO Materials

Plasma treatment is a universally used surface modification method for TCO materials, such as ITO, AZO, and FTO materials [98]. It enhances their surface energy, which leads to better bonding with other materials and improved adhesion. Moreover, it reduces particles and defects on the TCO material surface, resulting in increased transparency. Lastly, it enhances TCO materials’ electrical conductivity by altering their chemical composition and surface structure.

Plasma treatment can be divided into three steps. The initial stage involves plasma excitation, which refers to the procedure of exciting a gaseous species to a high-energy state via energy input. In plasma processing, an ionized plasma is generated by exciting and colliding gas molecules with electric arcs, microwave radiation, or radio frequency fields. The second stage involves the application of plasma to the surface of the material. This can be achieved by either spraying the plasma onto the material surface or immersing the material in the plasma. The third stage involves plasma dissociation. During the plasma dissociation phase, the impact of the process primarily depends on the interaction between the atoms, ions, or molecules created via dissociation and the surface of the TCO.

When plasma interacts with TCO materials, various physical and chemical reactions take place. Firstly, plasma surface treatment can efficiently eliminate organic residues and impurities from the TCO material surface, resulting in a pristine surface. Secondly, due to the surface chemistry that the plasma triggers, the TCO material’s surface energy level could be altered, thereby affecting its adhesion and wettability. These two changes enhance the quality and efficiency of the coating or deposition layer that follows. As the active particles within the plasma interact with the material surface, it is imperative to adjust the plasma parameters, such as power, time, and temperature, in order to achieve the most effective surface treatment outcome.

S. Major et al. [99] investigated the effect of hydrogen plasma treatment on ITO and FTO films. The results showed that the surfaces of ITO and FTO were reduced to produce elemental indium and tin, respectively. After annealing the plasma-treated films in air, the reduced surfaces were reoxidized, and the electro-optical properties were restored.

Compared with untreated TCO materials, plasma-treated TCO materials have the following advantages [100]: First, plasma treatment can change the morphology and chemical properties of the surface of TCO materials, thereby improving the connection between the TCO and the photocatalyst, resulting in improvement in the efficiency of PEC devices. Secondly, plasma treatment contributes to improved light absorption performance. By reducing optical defects on the TCO material surface, plasma treatment enhances light transmission and conductivity. This improvement is beneficial for enhancing the overall performance of devices such as solar cells and photodetectors. Thirdly, plasma treatment enhances the stability of TCO materials. The modified surface properties render them more stable and reliable, particularly when exposed to harsh conditions. This increased stability is advantageous for the long-term and reliable operation of TCO materials in various applications. Consequently, if the TCO device has low efficiency and deficiencies in light absorption performance and stability, it is possible to try to improve the performance with plasma treatment.

### 4.3. Other Treatments on TCO Materials

Hot isostatic pressing (HIP) is a high-temperature and high-pressure processing technology. In the HIP process, the material is compressed by simultaneously applying a high temperature of 100s to 2000 °C and an isostatic pressure of 10s to 200 MPa. That is, after the raw materials are pretreated, they are formed into dense products through the action of high-pressure inertia-guided shock waves and isostatic pressure at high temperatures. HIP processing eliminates micropores and microcracks in the material, improving its density and homogeneity. This results in shorter processing times and increased production efficiency. Additionally, HIP processing can sometimes reduce the need for high-performance materials, leading to cost savings.

For instance, Uematsu et al. [101] explored that high-purity and high-density tin dioxide doped with 10^20^ cm^−3^ antimony could be produced through hot isostatic pressing. They measured its electrical conductivity at temperatures ranging from 20 °C to 1200 °C in various atmospheres. They prepared ATO powders via pressureless sintering at 1400 °C and HIP at 1300 °C and found that the samples using HIP sintering have higher density and lower resistivity(10^−2^ Ω·cm).

**Figure 5:**
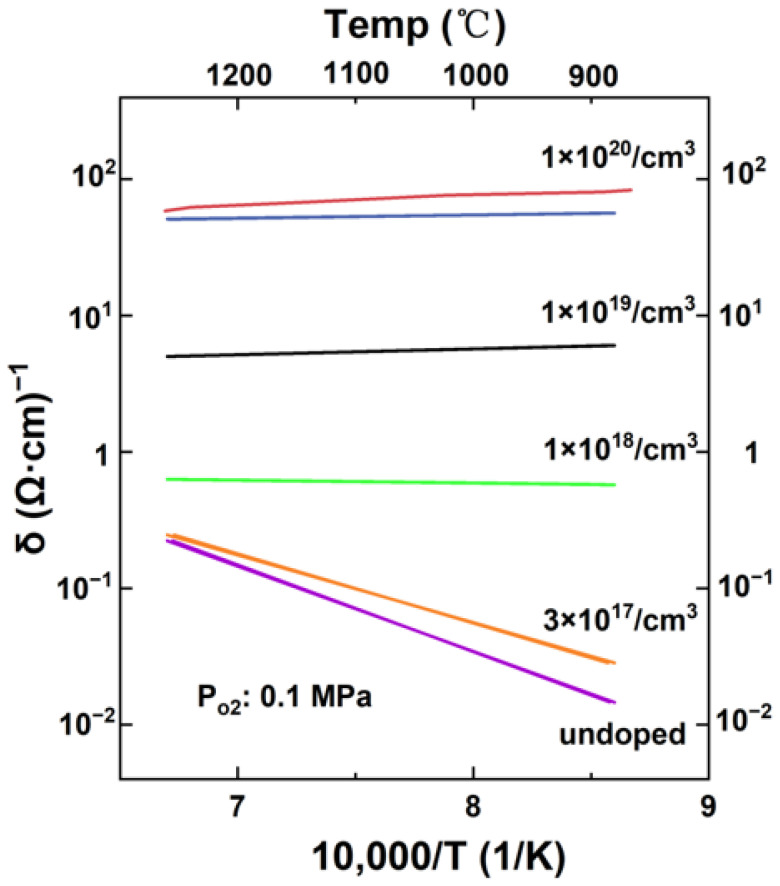
Effect of temperature on electrical conductivities. Solid lines show the results of antimony-doped, high-purity tin dioxide prepared via normal sintering. The line broken with solid dots shows the results of hot isostatic pressing. Reproduced with permission of Ref. [101], Copyright of © 1987 Journal of the American Ceramic Society.

Figure 5 illustrates the impact of temperature on the electrical conductivity of specimens, indicating a decrease in conductivity with an increase in temperature for both normally sintered and isostatically hot-pressed specimens. This decrease in conductivity is attributed to the reduced mobility of carriers that occurs as temperature increases. Notably, the specimen produced through isostatic hot pressing showed higher conductivity than the one sintered normally. However, it also demonstrated a quicker decrease in conductivity as the temperature increased. At higher temperatures, the conductivities of both types of specimens approached each other. Furthermore, similar results were obtained across different atmospheric conditions (within the bounds of experimental uncertainty). This suggests that HIP enables improved electrical conductivity at lower temperatures, but the temperature still constitutes an important factor that influences the conductivity of both normally sintered and isostatically hot-pressed specimens.

HIP treatment can improve the optical transmittance of TCO materials by reducing microcracks and surface defects. It can also optimize the grain size and grain boundary structure, leading to a decrease in electrical resistance and an improvement in electrical conductivity. Therefore, when TCO materials suffer micro-cracks, surface defects, uneven grain size, or grain boundary structure problems, we can try to improve its performance with HIP treatment to increase the transmittance and conductivity of the material, which will prolong its service life and improve the overall performance of the device.

Carbon nanotubes (CNTs), due to their extremely high mechanical performance and excellent electrical and thermal properties, have been regarded as ideal for the reinforcement phase of composites and have emerged with extensive applications in both electronic and optoelectronic domains [102]. The controlled growth of CNTs on appropriate substrates is crucial, especially on TCO substrates used in optoelectronic devices. While silicon substrates have traditionally been utilized in CNT synthesis due to their thermal and electrical stability, TCO substrates offer distinct advantages due to their unique optical and electrical properties, making them particularly attractive for nanodevices.

**Figure 6:**
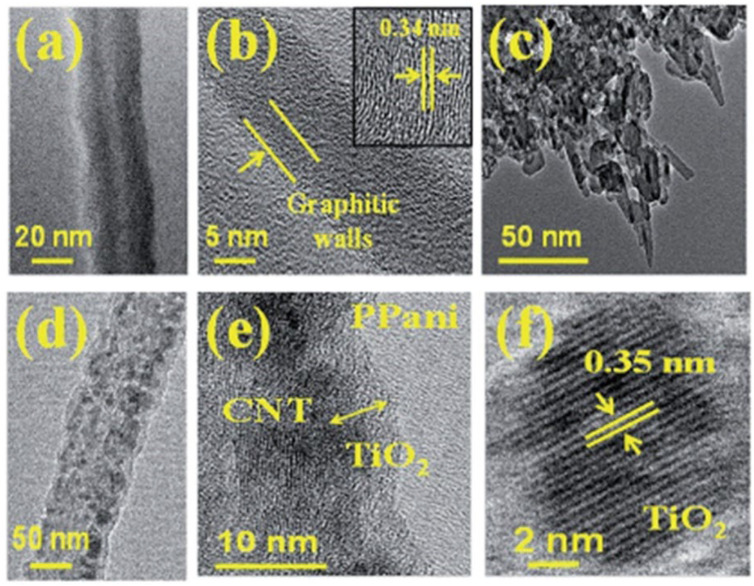
HRTEM images of (**a**) MWCNT and (**b**) magnified image of MWCNT showing the graphitic walls. In the inset, the corresponding lattice fringes of CNT are shown for (**c**) MWCNT-based hybrid composite, (**d**) MWCNT coated with TiO_2_, (**e**) MWCNT coated with TiO_2_ and Pani, and (**f**) lattice fringes of TiO_2_. Reproduced with permission of Ref. [103], Copyright of © 2014 RSC Advances.

Recent advancements have showcased successful controlled growth of CNTs on TCO substrates, marking a significant breakthrough [103]. HRTEM images in Figure 6a,b shows the morphology of a typical MWCNT and the corresponding graphitic walls. Further, Figure 6 also includes the HRTEM images of MWCNT based hybrid composite. A typical image containing CNT, TiO_2_ and PPani is shown in the first image (Figure 6c) which clearly distinguished the presence of MWCNTs coated with TiO_2_ and PPani. In the second image a magnied view of MWCNT coated with TiO_2_ is presented (Figure 6d) where the TiO_2_ nanoparticles are distributed on the walls of MWCNTs. Moreover, a magnified image of MWCNT/TiO2/PPani hybrid composite is distinctly shown in Figure 6e where the presence of TiO_2_ is found outside the graphitic walls of MWCNT. In addition, PPani matrix is uniformly distributed in the background to form a thin film. The presence of TiO_2_ in the hybrid composite material is inferred by direct visualization of lattice fringes in HRTEM micrographs. Figure 6f clearly shows the lattice fringes of TiO_2_ nanoparticle with 0.35 nm lattice spacing corresponding to the d values of (101) plane of anatase TiO_2_ nanoparticle. This achievement opens the door to developing hybrid structures that take advantage of the unique properties of both CNTs and TCO substrates. The synergistic combination of CNTs and TCO holds great promise for enhancing the performance of nanodevices, particularly in the realm of optoelectronics. This integration leverages the excellent electrical and thermal stability of CNTs with the distinctive optical and electrical characteristics of TCO substrates.

The growth of CNTs on TCO substrates demands precise control of process parameters, including the regulation of growth temperature, catalyst composition, and gas flow rate. Mahananda Baro et al. [90] delved into the direct synthesis of vertically aligned multi-walled CNTs (MWCNTs) through pulsed dc plasma-enhanced chemical vapor deposition (PECVD) on conducting indium tin oxide (ITO) substrates. The exceptional properties of CNTs, such as their high aspect ratio, electrical conductivity, and chemical stability, position them as highly attractive materials across a diverse range of electronic and optoelectronic applications. Composites of synthetic CNTs and TCOs exhibit improved photoelectrochemical properties compared to TCOs alone. CNTs efficiently collect and transport photogenerated electrons due to their excellent electrical and thermal properties, as well as their large specific surface area. This enhances the photoelectric conversion efficiency. Additionally, the introduction of CNTs improves light absorption and scattering, leading to enhanced light utilization. At the same time, CNTs improve the mechanical properties and stability of composites, allowing them to withstand harsh environments. Uday Narayan Maiti et al. [104] have found experimentally that doped carbon nanotubes can be incorporated into inorganic charge transport layers or other device components such as transparent conductive oxides (TCOs) to effectively enhance carrier mobility. They used N-doped carbon nanotubes to enhance the electron mobility of solution-processed ZnO electron transport layers while maintaining high optical transparency. To enhance the performance of TCO materials in photoelectrochemistry, researchers often synthesize CNT on TCO to form composites. This approach can improve mechanical strength, electrical conductivity, thermal conductivity, surface area, and charge transport. Composites of this kind combine the high transparency of TCO with the unique properties of CNT, making them suitable for specific applications.

Photonic crystals with a periodic structure play a crucial role in manipulating the propagation of photons, increasing the probability of light absorption. Specifically, photonic crystals made from transparent conductive oxide (TCO) materials exhibit unique properties, forming periodic structured materials with photonic forbidden bands. These bands enable control over the propagation of photons within the material. TCO photonic crystals can be prepared via various methods, such as constructing ordered macroporous or hollow sphere structures in the TCO material or incorporating periodic structures with photoconductive materials on the TCO material.

The potential applications of TCO photonic crystals extend to the fields of energy, optics, and photoconductivity. In optoelectronic devices, TCO photonic crystals offer the ability to adjust the position and width of the photonic forbidden band, optimizing light transmission and absorption. This, in turn, enhances the photoelectric conversion efficiency of optoelectronic devices. Moreover, TCO photonic crystals can be utilized in the fabrication of optical devices, modulators, filters, and more, presenting new possibilities for achieving more efficient optical devices.

**Figure 7:**
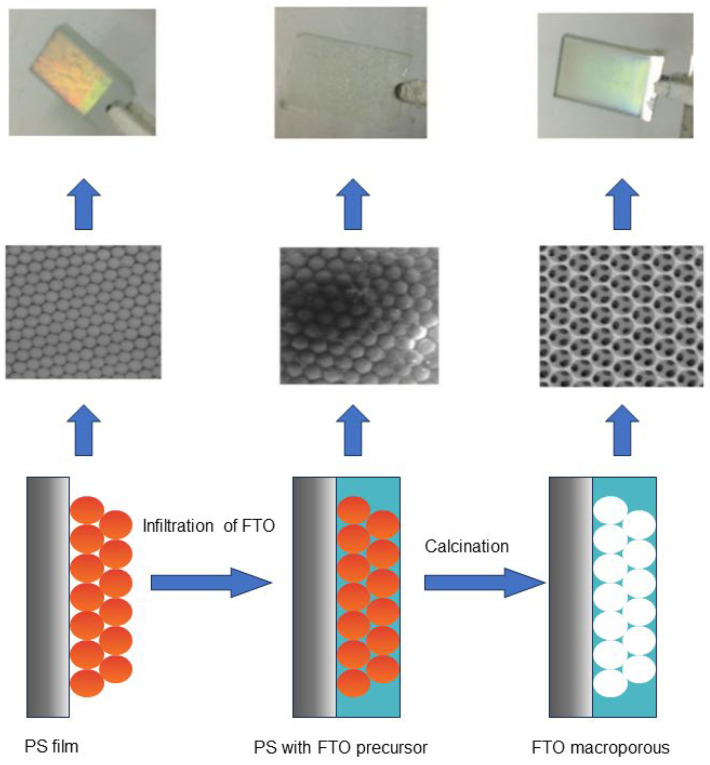
Schematic diagrams, SEM images, and photographs of the template polystyrene sphere film deposited on a FTO glass substrate (left); template polystyrene film infiltrated with FTO precursor (middle); and the mac-FTO obtained by removal of template polystyrene sphere film with calcination in this work. Reproduced with permission of Ref. [105]; this work © 2 by He Wen, Bing Wang, Wenbo Xiao, Xiao Liu, Yiming Wang, Menglong Zhang, and Haowei Huang is licensed under Attribution-NonCommercial-ShareAlike 4.0 International.

Zhang et al. [105] have developed a method to fabricate high-quality photonic TCO films, including macroporous FTO and hollow sphere AZ6O. High-quality optical and conductive mac-FTO films were obtained by optimizing the synthesis and processing conditions, as shown in Figure 7. The formation of films on photocatalysts such as CdS, C_3_N_4_, and Fe_2_O_3_ resulted in significant enhancement of the photocurrent at structured TCO electrodes. Among them, the photocurrent density of CdS@mac-FTO under visible light (>420 nm) reached 9 mAcm^−2^, which is better than the results of CdS working on structured electrodes previously reported in the literature. In addition, the newly developed hs-AZO can also support up to 7.8 mAcm^−2^ photocurrent after CdS coating. Both FTO and AZO exhibited significant photocurrent enhancement compared to planar FTO analogues. The photocurrent density of structured TCO electrodes can be further enhanced by increasing the surface area and loading more photoactive materials. Wang et al. [106] fabricated a photoelectrode using a photonic-crystal fluorine-doped tin oxide (PC FTO) film. The control CdS/planar-FTO photocurrent density decreased steadily as the angle of incidence decreased. At 0 degrees, the photocurrent density decreased by over 97% compared to 90 degrees. However, the photocurrent density of the CdS/PC FTO photoelectrode decreases similarly from 90°to 45°compared to the planar FTO. It only experiences an 8% loss of current density from 45° to 0°, which is significantly lower than the planar electrode’s loss of 28%. The results indicate that the PC electrode can significantly enhance the light harvesting capacity at certain incidence angles (below 45°). This enables the embedded photoactive material (CdS nanoparticles) to benefit from the properties of PC at different incidence angles, particularly at lower incidence angles.

Further investigation is required to combine various modification strategies, such as elemental doping, plasma treatment, thermal isostatic pressing, and carbon nanotube deposition on TCO substrates, to synthesize the advantages of multiple approaches and improve the properties of TCO materials. Future experiments should aim to explore the effects of combining different modification techniques and determining the optimal treatment sequence and parameters. It is also important to investigate the interactions between modification techniques and their effects on the microstructure and macroscopic properties of TCO. Additionally, the physical and chemical properties of the modified TCO should be analyzed. The modified TCOs are evaluated for their properties, such as electrical conductivity, light transmission, mechanical strength, and chemical stability, to ensure they meet the requirements of specific applications.

## 5. Conclusions and Future Prospect

In recent years, TCO materials have garnered considerable attention owing to their unique properties, making them highly suitable for diverse applications. Notably, they play a crucial role as substrate materials supporting PEC reactions, converting solar energy into chemical energy. Despite significant research advancements in modifying TCO materials for PEC reactions, their effectiveness falls short of meeting the requirements for large-scale industrialization. Addressing this challenge necessitates focused efforts in several key areas for future research:

1. Enhancing transparency and conductivity: Further research is imperative to improve the transparency and conductivity of TCO materials through cost-effective, simple, efficient, and environmentally friendly methods. Current production processes involve expensive materials and intricate procedures, including high-temperature and high-pressure conditions, resulting in elevated production costs. Additionally, ensuring stability under harsh conditions such as acid and alkali corrosion, elevated temperatures, and high moisture levels remains a critical challenge;
2. Formulating advanced theories: Advanced theories are essential to investigate reaction mechanisms and establish a comprehensive understanding of the structure–property relationships and carrier transfer mechanisms in TCO materials. Balancing performance indicators such as transparency, electrical conductivity, and mechanical strength during production is crucial but challenging. Developing advanced theories to customize the preparation process is vital for scaling up production;
3. Integration of multiple modification strategies: Combining various modification strategi0es, including element doping, plasma treatment, hot isostatic pressing, and carbon nanotube depositing on TCO substrates, holds significant promise. These strategies aim to enhance conductivity and transparency, elevate the rate of PEC reactions, and contribute to environmentally friendly practices, aligning with global sustainability goals.

In conclusion, future research efforts should prioritize the aforementioned improvement strategies for TCO materials. Continuous optimization in these areas is anticipated to provide a fresh perspective on the development of PEC devices, facilitating the functional utilization of efficient energy conversion and contributing to environmental pollution containment. The pursuit of these advancements underscores the transformative potential of TCO materials in advancing sustainable energy solutions.

## Figures and Tables

**Table 1:** Basic values of various TCO materials.

TCO Material	Transparency	Conductivity	Thermal Stability	Chemical Stability	E_g_
Fluorine-doped tin oxide (FTO)	High (80–90%)	Good ($10^{-4}\sim 10^{2}\;\Omega^{-1}$cm^−1^) [14]	Excellent	Relatively stable to strong acids and alkalis	Approx. 3.5–4 eV [15]
Indium tin oxide (ITO)	High (80–90%)	Good ($10^{-4}\sim 10^{2}\;\Omega^{-1}$cm^−1^) [16]	Ordinary	Easily corroded under strong acids and alkalis	Approx. 3.5–4 eV [17]
Indium zinc oxide (IZO)	High (80–90%)	$\mathrm{Good}\;(10^{-4}\sim 10^{2}\;\Omega^{-1}$cm^−1^) [18]	Excellent	Easily corroded under strong acids and alkalis	Approx. 3.5–4 eV [19]
Zinc oxide (ZnO)	High (80–90%)	$\mathrm{Fair}\;(10^{-4}\sim 10\;\Omega^{-1}$cm^−1^) [20]	Excellent	Corrosion-resistant to strong acids and alkalis	Approx. 3.3 eV [21]
Titanium dioxide (TiO_2_)	Medium (60–80%)	$\mathrm{Poor}\;(10^{-8}\sim 10^{-3}\;\Omega^{-1}$cm^−1^) [22]	Excellent	Relatively stable to strong acids and alkalis	Approx. 3.0–3.2 eV (anatase), approx. 3.7 eV (rutile) [23]
Indium oxide (In_2_O_3_)	High (80–90%)	$\mathrm{Good}\;(10^{-4}\sim 10^{2}\;\Omega^{-1}$cm^−1^) [24]	Excellent	Corrosion-resistant to strong acids and alkalis	Approx. 2.8–3.0 eV [25]
Gallium-doped zinc oxide (GZO)	High (80–90%)	$\mathrm{Good}\;(10^{-4}\sim 10^{2}\;\Omega^{-1}$cm^−1^) [26]	Excellent	Easily corroded under strong acids and alkalis	Approx. 3.3 eV [27]
Aluminum-doped zinc oxide (AZO)	High (80–90%)	$\mathrm{Fair}\;(10^{-4}\sim 10\;\Omega^{-1}$cm^−1^) [28]	Excellent	Corrosion-resistant to strong acids and alkalis	Approx. 3.3 eV [29]

**Table 2:** Doping of other TCO materials.

TCO Material	Transparency	Conductivity	Thermal Stability	Chemical Stability
F-doped ZnO	High (>90%)	$10^{-4}\sim 10^{-6}\;\Omega^{-1}$cm^−1^ [86]	High	Medium
Si-doped ZnO	High (>90%)	$10^{-4}\sim 10^{-6}\;\Omega^{-1}$cm^−1^ [87]	High	Medium
Cl-doped ZnO	High (>90%)	$10^{-4}\sim 10^{-6}\;\Omega^{-1}$cm^−1^ [88]	High	Medium
Y-doped ZnO	High (>90%)	$10^{-4}\sim 10^{-6}\;\Omega^{-1}$cm^−1^ [89]	High	Medium
Zn-doped In_2_O_3_	High (>90%)	$10^{-4}\sim 10^{-6}\;\Omega^{-1}$cm^−1^ [90]	High	Medium
Mo-doped In_2_O_3_	High (>90%)	$10^{-4}\sim 10^{-6}\;\Omega^{-1}$cm^−1^ [91]	High	Low
Sb-doped SnO_2_	High (>90%)	$10^{-4}\sim 10^{-6}\;\Omega^{-1}$cm^−1^ [92]	High	Low
Ti-doped SnO_2_	High (>90%)	$10^{-4}\sim 10^{-6}\;\Omega^{-1}$cm^−1^ [93]	High	High
Nb-doped SnO_2_	High (>90%)	$10^{-4}\sim 10^{-6}\;\Omega^{-1}$cm^−1^ [94]	High	High
Nb-doped TiO_2_	High (90%)	Non-conductive~$10^{-4}\Omega^{-1}$cm^−1^ [95]	High	Medium
Ga-doped CdO	High (93%)	$10^{-3}\sim 10^{-4}\;\Omega^{-1}$cm^−1^ [96]	Medium	High
Co-doped CdO	Medium (85%)	$10^{-2}\sim 10^{-3}\;\Omega^{-1}$cm^−1^ [97]	Medium	Medium

## Data Availability

No new data were created or analyzed in this study. Data sharing is not applicable to this article.
